# Clinical applications and challenges of CD40/CD40L signaling regulation in autoimmune diseases

**DOI:** 10.3389/fimmu.2026.1830312

**Published:** 2026-05-18

**Authors:** Yangyang Man, Xiaoni Chen, Yi Liu, Biao Zhang, Jiahua Hu, Xianliang Hou

**Affiliations:** 1 Laboratory Center, Guangxi Key Laboratory of Metabolic Reprogramming and Intelligent Medical Engineering for Chronic Diseases, the Second Affiliated Hospital of Guilin Medical University, Guilin, China; 2 Department of Central Laboratory, Shenzhen Hospital, Beijing University of Chinese Medicine, Shenzhen, Guangdong, China

**Keywords:** autoimmune disease, CD40, CD40L, rheumatoid arthritis, Sjögren’s syndrome, systemic lupus erythematosus, targeted therapy

## Abstract

The CD40–CD40L axis is a central costimulatory pathway that links innate and adaptive immunity and contributes to autoimmune inflammation. However, CD40 signaling does not operate in the same way across cell types, and these differences are relevant to both therapeutic efficacy and safety. In this review, we discuss the molecular features of CD40 and CD40L, the TRAF-dependent signaling pathways activated downstream of CD40, and the distinct cellular responses observed in B cells, dendritic cells, and macrophages. We also examine how dysregulated CD40/CD40L signaling contributes to key pathological features of Rheumatoid arthritis, Systemic lupus erythematosus, and Sjögren’s syndrome, including ectopic germinal center reactions, pathogenic autoantibody production, and chronic tissue inflammation. Platelet-derived CD40L and CD40 expression on vascular cells may also help explain the thromboembolic complications observed with early CD40/CD40L-targeted biologics. Current evidence suggests that safer therapeutic targeting of this pathway will require greater selectivity, particularly with respect to cell-specific signaling and Fc-mediated adverse effects.

## 1 Introduction

The CD40/CD40L signaling pathway links innate and adaptive immune responses. Initially recognized primarily as a T cell–B cell costimulatory pair, emerging research has expanded its significance across a broader array of immune and non-immune cell types. CD40, a type I transmembrane receptor of the TNF receptor superfamily, is constitutively or inducibly expressed on various cell types, including B cells, dendritic cells (DCs), macrophages, and a variety of endothelial and epithelial cells. Its ligand, CD40L (CD154), is predominantly expressed on activated CD4^+^ T cells and platelets. It exists in both membrane-bound and soluble forms, both of which retain the capacity to activate CD40 signaling and initiate downstream immune responses.

Despite its well-documented role in immune modulation, the cell type-specific organization of CD40 signaling and its context-dependent regulation remain incompletely understood. In autoimmune diseases such as rheumatoid arthritis (RA), systemic lupus erythematosus (SLE), and Sjögren’s syndrome (SS), chronic overexpression of CD40 and CD40L drives aberrant germinal center-like reactions, sustains pathogenic autoantibody production, and exacerbates tissue inflammation. Although numerous therapeutic agents targeting the CD40/CD40L axis have been developed, early-phase trials have highlighted significant safety challenges, particularly thromboembolic events associated with Fc-competent anti-CD40L antibodies. These findings suggest that therapeutic targeting of CD40/CD40L signaling will require greater selectivity in order to improve safety without compromising efficacy.

This review focuses on three related aspects of the CD40/CD40L axis. First, we examine the molecular architecture and expression patterns of CD40 and CD40L. Second, we detail the TRAF-dependent organization of downstream signaling modules, including NF-κB, MAPK, and PI3K/Akt, and explore how these pathways are interpreted in a cell-specific manner in B cells, DCs, and macrophages. Finally, we discuss the pathogenic contributions of CD40/CD40L signaling in RA, SLE, and SS, and evaluate emerging therapeutic strategies aimed at targeting this axis. By explicitly linking signaling logic to disease mechanisms, we aim to provide a rational framework for the development of safer, next-generation CD40-targeted therapies.

## 2 Molecular structure and expression of CD40/CD40L

### 2.1 CD40/CD40L structure

The CD40–CD40L pathway is often introduced as a co-stimulatory axis linking innate and adaptive immunity. However, its biological output is not uniform. Instead, it varies across cell types and disease settings, making the structural features and expression patterns of CD40 and CD40L particularly relevant to the cell-specific signaling and pathological roles examined later in this review. CD40, a type I transmembrane protein classified within the TNF receptor superfamily, operates as a signaling scaffold by recruiting TNF receptor-associated factors (TRAFs) via discrete motifs in its cytoplasmic tail upon ligand engagement, as it inherently lacks intrinsic enzymatic activity (1, 2). Engagement of CD40 by its ligand CD40L promotes receptor oligomerization and initiates downstream signaling programs (1, 3), which are discussed in detail in Section 2.

CD40L, a member of the TNF family, is primarily found on the surface of activated CD4^+^ T cells and platelets (1, 4), with its presence noted in both membrane-anchored and soluble configurations. The soluble variant arises chiefly through proteolytic cleavage—and to a minor degree via alternative splicing—yet both isoforms maintain the capacity to engage CD40 and initiate immune responses (1, 5). Platelet-derived soluble CD40L provides a mechanistic link between immune activation and thromboinflammatory processes, offering context for safety considerations in CD40L-targeted interventions (3, 4, 6). These structural features are relevant not only to receptor activation, but also to the distinct biological effects of CD40/CD40L signaling across cell types and disease settings.

### 2.2 Membrane-bound vs soluble forms of CD40/CD40L

#### 2.2.1 Agonistic properties of sCD40L

The CD40–CD40L axis exists both in membrane-bound forms (mCD40, mCD40L) and, through proteolytic shedding, in soluble forms (sCD40, sCD40L) (1). Membrane-bound CD40L is a typical contact-dependent costimulatory molecule, predominantly expressed by activated CD4^+^ T cells and platelets (1, 3). By engaging CD40 on B cells, dendritic cells and other antigen-presenting cells, it triggers TRAF-dependent signaling and thereby regulates humoral immunity and inflammatory responses (1). When mCD40L is cleaved at the cell surface by ADAM family metalloproteinases, soluble CD40L (sCD40L) is released (1, 3). This soluble form retains the TNF homology domain and predominantly exists as a trimer, although higher-order oligomeric or aggregated forms can also occur and are generally less abundant. It retains the ability to bind CD40 and function as a biologically active ligand (1, 7).

In most settings, sCD40L acts as an agonist that amplifies CD40 signaling: it binds CD40 on endothelial cells, smooth muscle cells and monocytes/macrophages, activates the NF-κB and MAPK pathways, and induces the expression of adhesion molecules, chemokines and matrix metalloproteinases, thereby promoting leukocyte adhesion, vascular inflammation and plaque instability (1, 3). In the cardiovascular system, sCD40L also interacts with integrins on platelets and leukocytes to enhance platelet–leukocyte aggregation and thrombus formation (3, 8).

#### 2.2.2 Modulatory roles of sCD40L and sCD40

The effects of sCD40L appear to depend on its oligomerization state and concentration (7, 9). *In vitro* studies have shown that different oligomerization states and concentrations of sCD40L differ in their ability to cross-link CD40 and activate downstream signaling: highly multimeric forms of soluble CD40L are more effective than simple trimers in inducing B-cell activation and proliferation, whereas at lower-order oligomerization or relatively low concentrations, sCD40L-triggered CD40 signaling is markedly attenuated (7, 9, 10). In this context, low-oligomer sCD40L may act mainly by competitively occupying CD40 binding sites and thereby partially dampening the strong cell–cell contact signals mediated by membrane-bound CD40L (7, 9). Currently, evidence remains limited as to whether low-oligomer sCD40L functions as a bona fide antagonist *in vivo*. It may therefore be more appropriate to view this effect as partial attenuation of excessive CD40 activation rather than complete antagonism (7, 9). In parallel, the soluble form of CD40 (sCD40) is considered a decoy receptor that can bind CD40L in the circulation and block its interaction with membrane CD40, and in patients with atherosclerosis, chronic kidney disease and other inflammatory disorders, elevated sCD40 levels are thought to reflect endogenous negative feedback regulation of the CD40/CD40L axis (11–13). Taken together, under different oligomerization states and microenvironmental conditions, sCD40L and sCD40 may cooperate to regulate the magnitude of CD40 signaling (7, 11).

#### 2.2.3 Clinical correlations of sCD40L

A substantial amount of clinical data shows that sCD40L levels are significantly linked to disease activity (5, 14, 15). Patients with unstable angina and acute coronary syndromes show significantly higher plasma sCD40L levels, which are associated with a greater risk of major adverse cardiovascular events, positioning it as a potential biomarker for platelet-driven thromboinflammatory conditions (14, 15). In systemic lupus erythematosus and various autoimmune disorders, increased levels of sCD40L have been linked to disease activity markers, inflammatory indicators, and the extent of organ involvement (5, 16). Additionally, reduced methylation at critical CpG sites within the X-chromosome CD40L promoter in CD4^+^ T cells is associated with higher SLEDAI scores. This suggests that both CD40L overexpression and its soluble form play a role in the continuous activation of the disease (17). Taken together, these lines of evidence indicate that sCD40L is not only an important pathological effector molecule within the CD40 axis but also a potential humoral biomarker reflecting the activity of cardiovascular and autoimmune diseases (5, 14–17). The distinction between membrane-bound and soluble forms is therefore relevant not only to immune regulation, but also to disease pathogenesis and therapeutic targeting.

### 2.3 Expression patterns of CD40 and CD40L

#### 2.3.1 Expression on immune cells

The presence of CD40 can be detected on various immune and non-immune cells, including B cells, dendritic cells (DCs), macrophages, and endothelial cells, with its expression levels often upregulated upon exposure to inflammatory signals (18, 19). On B cells, this molecule is essential for facilitating T cell-dependent immune responses; its engagement with CD40L derived from activated T cells drives B-cell expansion and subsequent antibody production (1, 20). In contrast, CD40L is mainly expressed on activated CD4^+^ T cells, where its expression is strictly governed by the activation status of the T cell and the nature of the immunological challenge (5, 18).

#### 2.3.2 Platelets, vasculature and systemic inflammation

CD40L is also expressed on platelets, and upon platelet activation, large amounts of soluble CD40L (sCD40L) are released. Soluble CD40L can act on endothelial cells, promote immune cell recruitment, and contribute to vascular inflammation (3, 21). Elevated levels of soluble CD40L have been demonstrated in systemic autoimmune diseases, such as SLE, where it is considered an important mediator of disease activity (7, 22). Platelets are one of the major sources of sCD40L, and the large amounts of sCD40L released upon platelet activation contribute to systemic inflammation and the development of vascular disease (3, 4). The expression of CD40L on platelets is therefore closely associated with immune responses in autoimmune diseases and cardiovascular disorders (3, 4, 7). These expression patterns suggest that CD40/CD40L signaling should not be viewed as a uniform pathway. Instead, its biological consequences are likely to depend on the cellular context in which receptor–ligand engagement occurs, a point that is relevant to both autoimmune pathogenesis and therapeutic targeting.

## 3 CD40/CD40L signal transduction mechanisms

### 3.1 Binding characteristics of the CD40 cytoplasmic tail with TRAFs

#### 3.1.1 TRAF-binding motifs on CD40

Signaling via CD40 is primarily transduced through its recruitment of TNF receptor-associated factors (TRAFs), a necessity arising from its short cytoplasmic domain which possesses no inherent kinase function (23, 24). Comprising TRAF1 to TRAF6, the TRAF family is characterized by a highly conserved C-terminal TRAF domain in each member, which plays an essential role in recognizing and docking with specific amino acid motifs within the cytoplasmic regions of TNFR family proteins (24, 25). Except for TRAF1, the other TRAF proteins also include an N-terminal RING finger domain and several zinc finger domains, which are essential for activating downstream signaling pathways. Removal of these domains in TRAF mutants can lead to a dominant-negative effect, disrupting normal TRAF-mediated signaling (26).

**Figure 1:**
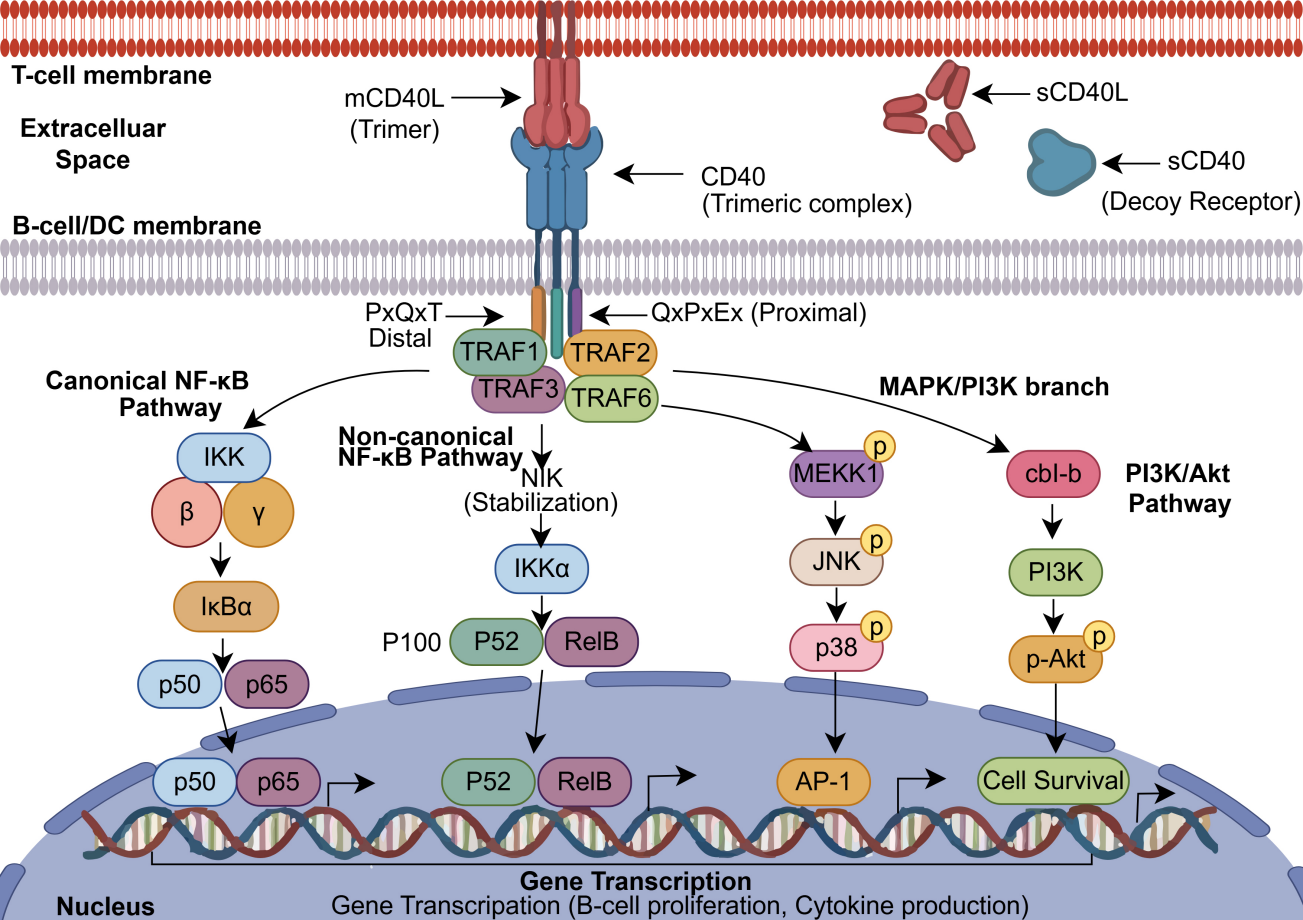
CD40–CD40L-mediated signal transduction. Upon engagement by membrane-bound trimeric CD40L, CD40 recruits TRAF proteins to its cytoplasmic PxQxT and QxPxEx motifs, thereby initiating the canonical and non-canonical NF-κB pathways, the MAPK module (JNK/p38), and the PI3K/Akt pathway. These signaling cascades collectively regulate gene transcription associated with cell survival, proliferation, and cytokine production. The figure also illustrates the soluble forms sCD40L and sCD40, with sCD40 shown as a soluble decoy receptor in the extracellular space.

Within the CD40 cytoplasmic tail, a canonical binding site shared by TRAF1, TRAF2, and TRAF3 resides in the membrane-distal region and is defined by a core PxQxT motif (27, 28). Disruption of this PxQxT motif through mutation abolishes the recruitment of TRAF2 and TRAF3 to the CD40 tail, leading to a pronounced impairment in the downstream activation of NF-κB, JNK, and p38 MAPK signaling cascades (27). Beyond this region, a non-canonical TRAF2 binding site is present in the C-terminal portion of the CD40 tail, which can partially sustain NF-κB activation even when the PxQxT region is compromised, thereby contributing to the regulation of various B-cell functions (28, 29). The TRAF6 binding site is located in a membrane-proximal QxPxEx sequence within the CD40 cytoplasmic tail; through this motif, TRAF6 is recruited to participate in NF-κB and PI3K/Akt signaling (27, 30). The structural arrangement of these binding motifs and the subsequent divergent signaling cascades they initiate are summarized in **Figure 1**. These binding motifs and their downstream signaling functions have been extensively characterized in earlier mechanistic studies and remain the canonical framework for understanding CD40 signaling (23, 27–30).

#### 3.1.2 Dynamic regulation by TRAF1

Upon CD40L engagement, trimeric CD40L induces conformational changes in CD40 that expose these TRAF binding motifs and enable multivalent interactions (31, 32). Crystallographic analyses have demonstrated that trimeric TRAF2 can simultaneously bind three CD40 cytoplasmic tails, forming higher-order oligomeric complexes (32). The extent of further oligomerization influences the recruitment of lower-affinity TRAFs (such as TRAF1 and TRAF6), as well as additional adaptor proteins and kinases, thereby shaping both the intensity and qualitative characteristics of CD40 signaling (31, 33). This fine-tuned regulation, determined by the specific combination of binding motifs and the degree of receptor–TRAF oligomerization, is considered a key structural basis for the diverse biological effects elicited by different CD40 monoclonal antibodies or ligand formats (34, 35).

Furthermore, TRAF1 predominantly plays a regulatory role in CD40 signaling (36, 37). CD40 activation can upregulate TRAF1 expression, and TRAF1 binds to a region on the CD40 cytoplasmic tail that partially overlaps with the TRAF2 binding site, thereby influencing both the recruitment and degradation of TRAF2 (36, 37). In TRAF1-deficient B cells, TRAF2 recruitment to CD40 is reduced and TRAF2 is more prone to degradation (36). In B cells with combined deficiency of TRAF1 and TRAF2, canonical NF-κB activation is markedly impaired, indicating that TRAF1 cooperatively participates in downstream CD40 signal transduction by regulating the stability and subcellular localization of TRAF2 (36). This fine-tuned regulation of TRAF protein recruitment and stability lays the molecular foundation for the selective amplification or suppression of CD40 signaling in different immune microenvironments, and also provides a structural explanation for its aberrant sustained activation under autoimmune conditions (31, 37).

### 3.2 NF-κB pathways: canonical and non-canonical

#### 3.2.1 Canonical NF-κB signaling

A central downstream effect of CD40 engagement is the triggering of NF-κB signaling. Via TRAF-dependent mechanisms, CD40 stimulates both the canonical and non-canonical NF-κB pathways. In the canonical NF-κB pathway, TRAF2 and TRAF6 function as critical upstream adaptor proteins (27, 38, 39). Upon the binding of CD40L, TRAF2 and TRAF6 are directed to the cytoplasmic tail of CD40, where they recruit and activate the IκB kinase (IKK) complex (33, 39). This complex is composed of the catalytic subunits IKKα and IKKβ, along with the regulatory subunit IKKγ/NEMO (40). Activated IKK phosphorylates IκB proteins, leading to their ubiquitination and subsequent degradation by the proteasome. Consequently, NF-κB heterodimers such as p50/p65 and p50/c-Rel are freed from the cytoplasm and move into the nucleus, where they initiate the transcription of numerous target genes related to inflammation, cell survival, and the expression of co-stimulatory molecules (31, 40). TRAF1 stabilizes TRAF2 and promotes its proper recruitment, making it essential for the activation of canonical NF-κB signaling through CD40 (36).

#### 3.2.2 Non-canonical NF-κB signaling

Activation of the non-canonical NF-κB pathway relies on the association of NF-κB-inducing kinase (NIK) with IKKα (41, 42). Under basal conditions, a complex composed of TRAF2, TRAF3, and cIAP1/2 constitutively promotes the ubiquitination and subsequent degradation of NIK, thereby suppressing its unstimulated activation of this pathway (38, 42, 43). Upon CD40/CD40L engagement, TRAF2 and TRAF3 are displaced from the NIK complex and recruited to the cytoplasmic tail of CD40 (38, 43). TRAF2 undergoes self-ubiquitination and degradation via its E3 ligase activity, while TRAF3 is degraded in a cIAP1/2-dependent fashion (38, 41, 42). This release of NIK from constant suppression allows NIK to accumulate and activate IKKα. Once activated, IKKα cleaves p100 to generate p52, which subsequently dimerizes with RelB and migrates to the nucleus, where it governs the expression of genes implicated in lymphoid organogenesis, cell survival, and late-stage immune responses (38, 43).

#### 3.2.3 Dual and context-dependent roles of TRAF2

TRAF2 exerts dual functions in a context-dependent manner downstream of CD40 signaling (38, 44). Within mature B cells, this molecule is essential for CD40-driven canonical NF-κB (NF-κB1) activation, thereby promoting IKK-mediated proinflammatory responses (44). Supporting this, mechanistic investigations have demonstrated that phosphorylation of TRAF2 at Ser-11 is required to sustain full and prolonged IKK activation upon CD40 engagement (45). In contrast, together with TRAF3 and cIAP1/2, TRAF2 maintains constitutive ubiquitination and degradation of NIK, thereby restraining non-canonical NF-κB signaling (38, 42, 43).

### 3.3 MAPK and PI3K/AKT signaling

#### 3.3.1 MAPK modules downstream of CD40

Upon CD40 ligation, adaptor proteins of the TRAF family couple CD40 to multiple downstream signaling cascades, most notably the mitogen-activated protein kinase (MAPK) modules and the phosphoinositide 3-kinase (PI3K)/Akt pathway, in addition to NF-κB and PLCγ signaling (1, 31). Upon its recruitment to the CD40 cytoplasmic domain, TRAF2 promotes the activation of Jnk, p38, and Akt; accordingly, in TRAF2-deficient mouse embryonic fibroblasts and B cells, the induction of these signaling cascades following CD40 engagement is severely impaired (27, 46, 47). TRAF2 also interacts with the upstream kinase MEKK1, and in B cells lacking MEKK1, CD40 stimulation fails to fully activate JNk and p38, indicating that MEKK1 is a key mediator of CD40-dependent MAPK activation (47).

#### 3.3.2 PI3K/Akt and pro-survival signaling

In parallel, CD40 can induce the recruitment of a TRAF6/Cbl-b/c-Cbl/PI3K complex to its cytoplasmic tail, which leads to Akt phosphorylation (46, 48). In dendritic cells deficient in Cbl-b and stimulated *in vitro* with CD40L, Akt activation is ablated, suggesting that in DCs, CD40-induced Akt activation via PI3K is functionally separable from NF-κB signaling (48). Blocking PI3K after CD40 engagement abrogates Akt activation, confirming that Cbl-b–dependent recruitment of PI3K is required for Akt phosphorylation during CD40 signaling (48, 49).

From a physiological standpoint, blockade of PI3K severely compromises the survival of dendritic cells (DCs) (31, 50). The pro-survival effect of PI3K/Akt signaling is partly achieved by suppressing pro-apoptotic molecules such as caspase-9 and the Bcl-2–associated agonist of cell death (31, 51). A second pathway involves PI3K-dependent activation of mTOR, which subsequently up-regulates anti-apoptotic regulators, including cFLIPp43 (31). Taken together, these observations indicate that CD40-induced activation of MAPK and PI3K/Akt signaling through TRAF2, MEKK1, TRAF6 and Cbl-b not only shapes inflammatory responses but also delivers strong survival cues, particularly to DCs. Such survival and anti-apoptotic signals support the long-term maintenance of activated immune cells within inflammatory microenvironments and are regarded as an important molecular basis for the chronic persistence of autoimmune inflammation (1, 31).

### 3.4 Cell type-specific differences in CD40 signaling

#### 3.4.1 In B cells

Although the adaptor proteins involved in CD40 signaling are largely shared, the nature of the downstream signaling pathways exhibits marked differences among distinct cell types. Among these, TRAF3 has emerged as one of the most thoroughly characterized examples. Initial investigations suggested that TRAF3 acts mainly as a suppressor of NF-κB signaling downstream of CD40, with a particular emphasis on the non-canonical pathway (38, 52–54). Consistent with this notion, in B cells lacking TRAF3 or expressing dominant-negative TRAF3, enhanced JNK phosphorylation, increased NIK accumulation, and excessive activation particularly the non-canonical, and in some contexts also the canonical, NF-κB pathways have been observed, indicating that TRAF3 restrains these signaling pathways in B cells (52–56). In airway epithelial cells, TRAF3 has been shown to participate in CD40-mediated activation of canonical NF-κB, and interference with TRAF3 function impairs NF-κB nuclear translocation (57). By contrast, in endothelial cells, shear stress–induced upregulation of TRAF3 attenuates CD40-dependent endothelial activation (58). Thus, TRAF3 displays pronounced functional heterogeneity across cell types: in B cells, it acts as a key negative regulator of canonical and non-canonical NF-κB as well as JNK signaling (52–56); whereas in certain epithelial-derived or parenchymal cell contexts, TRAF3 has been associated with enhanced activation of TAK1-dependent inflammatory signaling, highlighting the cell type-specific functional diversity of TRAF3 (59).

#### 3.4.2 In dendritic cells

In dendritic cells (DCs), Cbl-b also exhibits a clear cell type–dependent regulatory pattern (46, 60). Upon CD40 ligation, CD40 recruits a TRAF6/Cbl-b/c-Cbl/PI3K complex, which in turn drives robust Akt phosphorylation (46, 49, 60). In Cbl-b–deficient DCs, CD40-induced Akt activation is almost completely abolished, suggesting that in this context Cbl-b functions as a positive regulator of the PI3K/Akt pathway and promotes DC survival (46, 49, 60).

By contrast, in B cells, the absence of Cbl-b leads to enhanced NF-κB and JNK signaling upon CD40 stimulation, accompanied by increased recruitment of TRAF2 to the cytoplasmic tail of CD40, suggesting that under these conditions, Cbl-b functions as a “brake” by restraining TRAF2-mediated signaling (61). Collectively, these observations reinforce the concept that the precise signaling cascades governed by Cbl-b in various cellular contexts are dictated both by the specific TRAF family members it engages and the prevailing cellular environment (60, 62).

#### 3.4.3 In monocytes and other APCs

Cell type specificity in CD40 signaling is also reflected in the utilization of Jak3 in monocytes and other antigen-presenting cells (APCs) (63, 64). Previous studies have demonstrated that a Jak3-binding domain resides within the membrane-proximal portion of the CD40 cytoplasmic tail (63). In these cells, CD40 activation induces Jak3 phosphorylation, and inhibition of Jak3 during CD40 stimulation blocks APC maturation (64, 65). Upon activation, Jak3 phosphorylates STAT5 and promotes its dimerization and subsequent nuclear translocation, which in turn drives the expression of inflammatory cytokines such as TNF-α and IL-6 (31, 64). By contrast, in resting primary B cells, CD40 crosslinking does not induce Jak3 phosphorylation, indicating that although Jak3 can bind to CD40, it does not form an effective downstream signaling cascade in this cell type (64). These observations suggest that Jak3-dependent CD40 signaling promotes inflammatory cytokine production and APC maturation in monocytes and other APCs, whereas this signaling branch appears to be functionally limited in resting B cells.

## 4 Role of CD40/CD40L in immune regulation

### 4.1 Role of CD40/CD40L in B-cell activation, germinal center responses, and antibody production

#### 4.1.1 CD40 signaling modules in B cells

CD40-mediated signaling plays an indispensable role in both the induction and persistence of T cell-dependent humoral immunity (1, 66). Through engagement with its ligand CD40L, CD40 activates multiple key signaling pathways that provide the central driving force for B-cell proliferation, differentiation, and functional maturation (1, 66, 67). The cytoplasmic tail of CD40 recruits members of the tumor necrosis factor receptor–associated factor (TRAF) family, including TRAF1, TRAF2, TRAF3, TRAF5, and TRAF6, thereby activating the canonical and non-canonical NF-κB pathways, mitogen-activated protein kinase (MAPK) pathways (JNk and p38), and the phosphatidylinositol 3-kinase/protein kinase B (PI3K/Akt) pathway (1, 31, 67). In parallel, CD40 signaling regulates the expression of apoptosis-related molecules such as Bcl-XL and c-FLIP, ultimately driving B-cell proliferation, immunoglobulin class-switch recombination, somatic hypermutation (SHM), and memory B-cell formation (1, 31, 68). These signaling outputs provide the molecular basis for the central role of CD40 in humoral immunity and help explain why dysregulated CD40 signaling can promote aberrant germinal center responses and pathogenic antibody production in autoimmune disease (1, 31, 66–68).

#### 4.1.2 CD40–CD40L interactions in T cell–dependent humoral responses

During T cell–dependent immune responses, antigen-stimulated B lymphocytes display peptide antigens on their surface MHC class II molecules to CD4^+^ helper T cells, thereby initiating cognate T–B interactions (20, 69). At this stage, CD40L on activated T cells binds CD40 on B cells and provides an essential costimulatory signal (1, 20, 70). Working in concert with B-cell receptor (BCR)–driven antigen signaling, this costimulation allows B cells to overcome anergy and to enter a phase of sustained proliferation and differentiation (20, 70). *In vitro* experiments have shown that CD40 ligation directly triggers intercellular adhesion, clonal expansion, differentiation, and immunoglobulin class-switch recombination in B cells (70–72). Within living organisms, this same engagement is essential for the formation and persistence of germinal centers (GCs), as well as for isotype switching and affinity maturation, thereby facilitating the production of memory B cells and long-lived plasma cells (66, 69, 73, 74). Interference with CD40–CD40L engagement, either by blocking antibodies or by genetic deletion, completely abolishes T cell–dependent humoral immune responses (70, 75, 76). Thus, CD40–CD40L engagement functions as a non-redundant checkpoint for productive T cell–dependent humoral immunity, and its dysregulation may lower the threshold for pathogenic germinal-center responses and autoantibody generation.

#### 4.1.3 CD40 control of germinal center selection and affinity maturation

Germinal centers represent the central sites for affinity maturation and functional selection of B cells, and CD40 signaling serves as a core regulator of the “selection–proliferation–affinity maturation” cycle within GCs (69, 77). Following somatic hypermutation, GC B cells display heterogeneous BCR affinities (69, 78). Only GC B cells (centrocytes) expressing high-affinity BCRs can efficiently capture antigen complexes displayed on follicular dendritic cells (FDCs) and receive sufficient survival signals through engagement of CD40 with CD40L expressed on follicular helper T (Tfh) cells (20, 77–79). In contrast, low-affinity or autoreactive B cells fail to obtain adequate CD40 signaling and are eliminated via Fas-dependent apoptotic pathways (80–82). As the principal source of CD40L within germinal centers, Tfh cells provide CD40-mediated signals that are not only essential for B-cell survival but also promote immunoglobulin class-switch recombination and somatic hypermutation, thereby supporting affinity maturation and the generation of high-affinity antibodies (83).

Beyond its role in supporting GC B-cell survival, CD40 signaling also shapes the qualitative output of the germinal center reaction. Subsequent studies have shown that the binding of distinct TRAF adaptor proteins to CD40 governs different phases of the germinal-center reaction. Loss of the TRAF6 docking site leads to defective antibody affinity maturation and reduced plasma cell formation, whereas combined mutations affecting both the TRAF2–TRAF3 and TRAF6 binding motifs entirely block germinal center formation. In such situations, early B-cell proliferation and initial Ig production are preserved, but later affinity maturation fails to occur (83). Together, these findings indicate that CD40 signaling not only supports germinal center B-cell survival, but also helps shape the quality and outcome of the germinal center response, including affinity maturation and plasma cell generation.

#### 4.1.4 Genetic and clinical consequences of CD40/CD40L deficiency

Genetic deficiency models further illustrate the non-redundant role of the CD40/CD40L axis in humoral immunity. In mice deficient in either CD40 or CD40L, functional germinal centers fail to develop, immunoglobulin class switching from IgM to IgG, IgA, or IgE is severely impaired, and immunological memory is not properly established (75, 84). As a result, these animals predominantly produce low-affinity IgM antibodies, highlighting the essential role of CD40/CD40L signaling in productive germinal center responses (75, 84). Comparable defects are also observed in humans. Among human populations, loss-of-function mutations within the CD40L gene lead to the development of X-linked hyper-IgM syndrome (85–87). In addition, CD40 deficiency can produce a related hyper-IgM immunodeficiency phenotype, further underscoring the requirement for intact CD40/CD40L signaling in human humoral immunity (88, 89). Because CD40–CD40L signaling is disrupted, affected patients have profound defects in T cell–dependent humoral immunity, typically showing very low serum levels of IgG, IgA and IgE, while IgM concentrations are normal or increased (85, 87, 90). Such patients cannot generate germinal centers, fail to develop memory B cells and lack class-switched protective antibodies, making them highly vulnerable to bacterial infections (85, 86, 89, 90). Viewed together, the murine deficiency models and the human hyper-IgM phenotype provide complementary evidence that intact CD40/CD40L signaling is indispensable for germinal center formation, immunoglobulin class switching, and the generation of durable humoral immune memory.

### 4.2 CD40-induced dendritic cell maturation and enhanced antigen presentation

#### 4.2.1 CD40-driven dendritic cell differentiation and phenotypic maturation

Enrichment and phenotypic analyses of dendritic cell subsets isolated from human peripheral blood, tonsillar tissue, and dermis have defined distinct populations in these compartments, and several of these, including epidermal Langerhans cells, have been shown to express functional CD40 (91–94). Experimental investigations have demonstrated that engagement of CD40 on CD34^+^ hematopoietic progenitor cells derived from umbilical cord blood, as well as on adherent monocytes from peripheral blood, stimulates their expansion and maturation into fully functional dendritic cells (95–97). In parallel, CD68^+^lin^−^ cells in peripheral blood have been identified as dendritic cell precursors (98), further supporting a critical role for CD40 and related signals in regulating dendritic cell development and maturation.

Activation of CD40 induces the formation of dendrite-like cellular protrusions, triggers phenotypic changes, and upregulates the expression of MHC class II molecules, CD25, CD58, CD80 (B7-1), CD86 (B7-2), and CD40L. Concurrently, engagement of CD40 drives the release of various cytokines—including TNF-α, IL-8, IL-10, and IL-12—along with chemokines such as MIP-1α, MIP-1β, and RANTES (97, 99, 100). Together, these changes enhance the activation status of dendritic cells and significantly increase their antigen-presenting capacity (97, 99).

#### 4.2.2 Cytokine production, IL-12 regulation and T-cell priming

A number of studies have shown that CD40/CD40L-dependent crosstalk between T lymphocytes and dendritic cells is crucial for the proper development of B-cell follicles and for mounting effective adaptive immune responses (1, 31, 101). Focusing on IL-12 induction, early work proposed that CD40 ligation by itself was enough to trigger IL-12 production. Later investigations, however, demonstrated that expression of the IL-12 p40 and p75 subunits in dendritic cells actually requires an additional signal delivered by antigen-specific Th1 cells in the form of IFN-γ. Notably, these Th1 cells are indistinguishable in terms of TCR clonotype and CD40L expression levels (106, 107). In contrast, Th2 cells fail to drive IL-12 synthesis and instead actively down-regulate its production (102).

In pathological *in vivo* settings, increased CD40 expression has been documented in diffuse large B-cell and other non-Hodgkin lymphomas, both on malignant B cells and within the tumor stroma, where it marks an active inflammatory and antigen-presenting microenvironment (103, 104). In autoimmune conditions like rheumatoid arthritis, the synovium contains abundant populations of mature antigen-presenting dendritic cells characterized by elevated expression of MHC class II and co-stimulatory molecules—including CD40—thereby promoting the sustained activation of autoreactive T cells within the inflamed joint (105, 106). Taken together, these findings indicate that CD40-dependent cytokine production in dendritic cells, particularly IL-12 in the appropriate inflammatory context, strengthens T-cell priming and favors sustained autoreactive T-cell activation in autoimmune lesions.

#### 3.2.3 Survival, migration and antitumor responses

CD40 cross-linking confers resistance to Fas-induced apoptosis in human dendritic cells (50), and CD40L similarly inhibits Fas/CD95-mediated apoptosis in blood-derived dendritic cells (107), thereby contributing to the maintenance of dendritic cell numbers required for effective antigen presentation. CD40L-induced dendritic cell maturation rapidly downregulates the expression of CCR1 and CCR5, while gradually upregulating CCR7 expression (108–110). This chemokine receptor switch enables mature dendritic cells to migrate to lymphoid organs, where they more effectively initiate T-cell responses (108–110).

In dendritic cells, CD40 engagement has also been linked to the induction of inducible nitric oxide synthase and the subsequent production of nitric oxide (101, 111). Although the precise consequences may depend on context, available evidence suggests that this response forms part of the activation program of CD40-stimulated DCs and may contribute to their capacity to support downstream T-cell responses, particularly under inflammatory or antitumor conditions (111). CD40/CD40L interactions participate in dendritic cell-mediated T-lymphocyte activation (1, 101). Moreover, CD40 cross-linking on dendritic cells is critical for the generation of protective antitumor immune responses, which depend on the enhanced antigen-presenting capacity of dendritic cells (112, 113). Taken together, these effects indicate that CD40 signaling supports dendritic cell survival, migration, and functional maturation, thereby prolonging antigen presentation and strengthening downstream T-cell activation. In inflammatory settings, such sustained dendritic cell activity may promote continued immune activation rather than transient protective responses.

### 4.3 Role of CD40 in macrophage activation and inflammatory amplification

#### 4.3.1 Monocyte and macrophage activation by CD40L

Human peripheral blood monocytes, as well as the monocytic cell line U937, upregulate CD40 mRNA and surface protein upon exposure to GM-CSF, IL-3, IFN-γ, or soluble CD23 (114, 115). Additionally, monocytes display constitutive expression of CD40 (114). The interaction between CD40 and CD40L is a critical trigger for contact-dependent activation of monocytes by CD4^+^ T cells (114, 116, 117). This process is bidirectional, as phenotypic changes in monocytes can further enhance and/or prolong T-cell activation and inflammatory responses (116).

Ligation of CD40 on monocytes/macrophages induces IL-12 expression (118, 119), while IL-12 in turn promotes CD40L expression on T lymphocytes (120), thereby forming a positive feedback amplification loop. Abnormal CD40 expression and signaling patterns in monocytes/macrophages have been reported in ultraviolet-irradiated skin and in the peripheral blood of HIV-1–positive patients (121, 122). Functionally, although early studies suggested that CD40 activation required cytokine costimulation (114), subsequent studies demonstrated that CD40 ligation alone is sufficient to induce the expression of TNF-α, IL-1, IL-6, and IL-8 in peripheral blood monocytes (116). The induction of IL-1β and TNF-α may depend on the MEK/ERK signaling pathway and can be antagonized by signals generated by IL-4 and IL-10 (123, 124). Together, these findings indicate that CD40/CD40L-driven monocyte and macrophage activation promotes a pro-inflammatory cytokine milieu that can amplify local immune responses, sustain leukocyte recruitment, and reinforce chronic inflammatory circuits. In autoimmune settings, this amplification is likely to contribute to persistent tissue inflammation and progressive tissue injury (123, 125).

#### 4.3.2 Matrix remodeling and thrombogenic potential

CD40L-positive T cells or soluble CD40L induce the expression of MMP-1, MMP-2, MMP-3, and MMP-9 in THP-1 cells, peripheral blood monocytes, and monocyte-derived macrophages (126–128). Through these matrix metalloproteinases, CD40/CD40L signaling in monocytes and macrophages contributes to extracellular matrix remodeling and tissue destruction in chronically inflamed tissues (126–128). Tissue factor expression in monocytes can be mediated by CD4^+^ helper T cells through a contact-dependent mechanism (129) and, in the context of CD40 ligation, is induced via an IL-10–resistant pathway (130), thereby enhancing the thrombogenic potential of inflamed vascular and perivascular sites. By driving both matrix degradation and tissue factor–dependent coagulation, CD40/CD40L-activated monocytes and macrophages link local inflammatory activation to structural tissue damage and a prothrombotic state (126, 129, 130).

### 4.4 CD40/CD40L in T-dependent humoral responses and immune memory

In addition to the cell-specific effects described above, CD40/CD40L signaling also plays a central role in coordinating T cell-dependent humoral responses. Upon T-cell receptor engagement, activated CD4^+^ T cells rapidly upregulate CD40L, which engages CD40 on B cells and antigen-presenting cells (66). This interaction provides an essential costimulatory signal for productive T cell–B cell collaboration and supports germinal center formation, class-switch recombination, affinity maturation, and the generation of memory B cells and long-lived plasma cells (66, 77, 131). In addition to these effects on B-cell responses, cross-linking of CD40L on T cells can also deliver costimulatory signals that influence T-helper differentiation and cytokine production (132, 133). Continued CD40-dependent communication also supports the maintenance and reactivation of humoral memory during secondary antigen exposure (77, 131). In chronic inflammatory and autoimmune settings, however, persistent CD40/CD40L activation may sustain pathogenic T–B collaboration and autoreactive humoral responses rather than protective immune memory (134).

### 4.5 Role of CD40/CD40L in the breakdown of immune tolerance

Building on the cell-type–specific roles outlined in Sections 3.1–3.4, CD40/CD40L signaling as a whole represents a key axis in the breakdown of immune tolerance (16, 31). Under physiological conditions, CD40-mediated costimulation supports productive T–B collaboration and adaptive immune responses (20, 31). However, persistent or dysregulated activation of this pathway lowers the threshold for autoreactive T- and B-cell activation and favors the survival and expansion of pathogenic clones (16, 31).

A broad range of experimental models support this view. CD40/CD40L interactions have been implicated in lupus-like nephritis, collagen-induced arthritis, experimental autoimmune encephalomyelitis and spontaneous autoimmune diabetes, among others (16, 135–139). In these settings, CD40-dependent activation of antigen-presenting cells and T cells promotes Th1/Th17 polarization, autoantibody production and inflammatory tissue injury (16, 31, 137, 138). Conversely, genetic disruption or antibody-mediated blockade of CD40L prevents disease onset or markedly attenuates pathology across multiple models, underscoring the non-redundant contribution of this pathway to autoimmune priming and effector function (135–139).

At the molecular level, studies in CD40L-deficient mice and adoptive transfer systems have shown that CD40L-dependent costimulation is required for optimal IFN-γ production by antigen-presenting cells and autoreactive T cells (140, 141). CD40 engagement further induces a panel of pro-inflammatory mediators—including IL-12, TNF-α and the costimulatory molecule CD80—that collectively reinforce Th1-biased responses and favor the persistence of autoreactive lymphocytes (101, 116, 118, 142). Together, these data indicate that CD40/CD40L signaling acts both upstream, by licensing autoreactive T cells, and downstream, by sustaining local inflammatory circuits in target tissues (16, 101, 140).

#### 4.5.1 Evidence from human autoimmune diseases and immunodeficiency

Human studies similarly support a central role for CD40/CD40L dysregulation in the breakdown of immune tolerance (16, 143–145). Across multiple autoimmune diseases, increased expression of CD40L on activated CD4^+^ T cells and upregulation of CD40 on B cells, monocytes and tissue-resident stromal cells have been reported, often correlating with disease activity and autoantibody titers. Prototypic examples include systemic lupus erythematosus and rheumatoid arthritis, where enhanced CD40/CD40L expression is accompanied by expanded germinal-center–like reactions and the production of high-affinity pathogenic autoantibodies (see Sections 4.1 and 4.2) (16, 143–145).

Complementary insights come from human immunodeficiency syndromes affecting this pathway. Patients with CD40L or CD40 deficiency develop hyper-IgM immunodeficiency, characterized by impaired class-switch recombination and profound defects in T cell–dependent antibody responses (88, 146). These conditions highlight that intact CD40/CD40L signaling is indispensable for normal humoral immunity. At the same time, epidemiological data linking hyper-IgM patients to an increased incidence of autoimmune manifestations, including rheumatoid arthritis, indicate that quantitative or qualitative alterations in CD40/CD40L function can destabilize immune homeostasis and predispose to loss of tolerance (146, 147).

**Figure 2:**
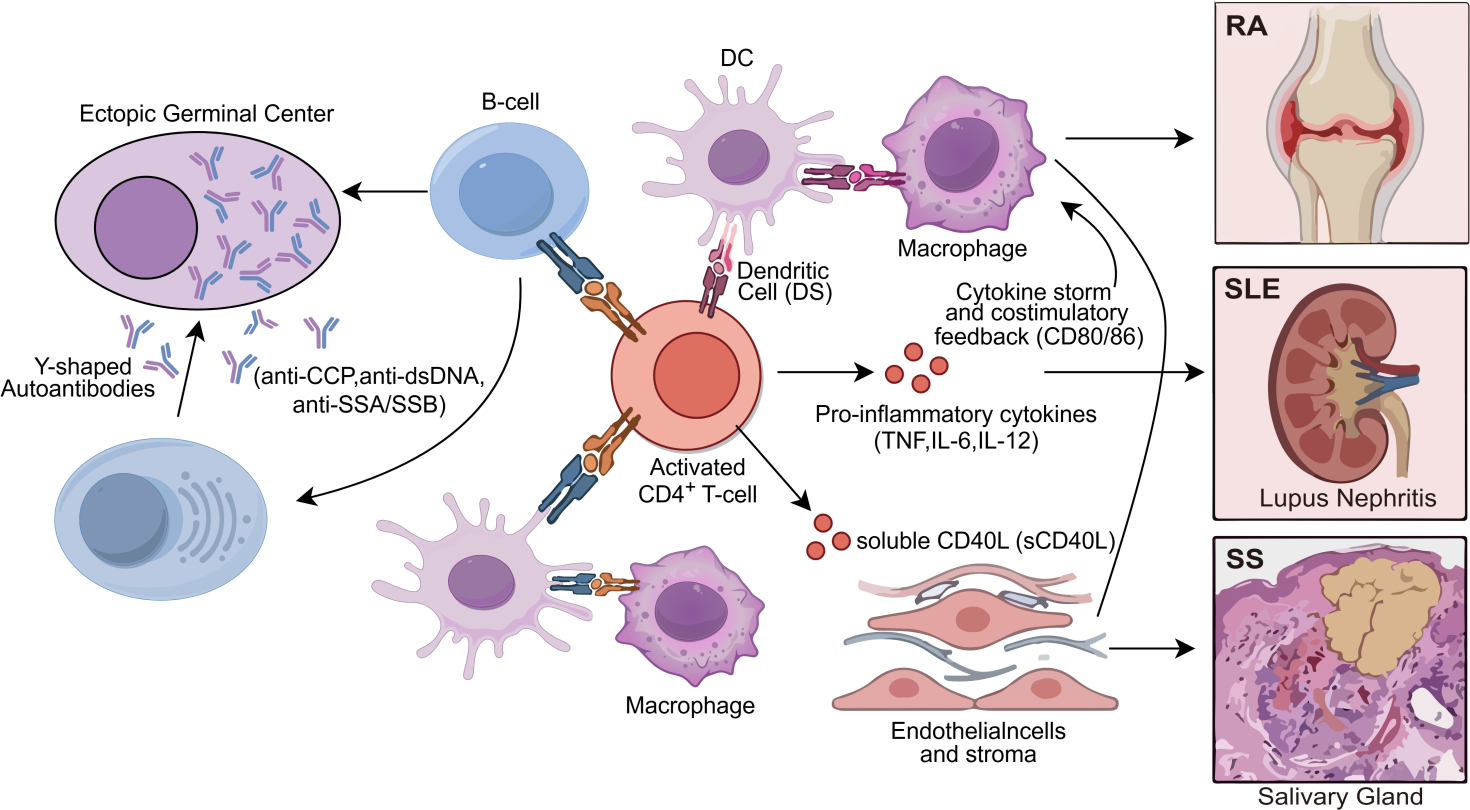
Pathogenic role of the CD40/CD40L axis in autoimmune diseases. Dysregulated interactions between activated CD4^+^ T cells and various effector cells (B cells, DCs, and macrophages) drive ectopic germinal center formation and pathogenic autoantibody production (e.g., anti-CCP, anti-dsDNA, anti-SSA/SSB). These processes result in organ-specific manifestations, including joint destruction in RA, lupus nephritis in SLE, and salivary gland inflammation in SS.

Taken together, converging evidence from experimental models, human autoimmune cohorts and rare immunodeficiency syndromes indicates that CD40/CD40L signaling occupies a critical checkpoint in immune tolerance (16, 88, 144). Once this checkpoint is breached, the pathway promotes autoreactive T–B collaboration, pathogenic autoantibody production and chronic tissue inflammation, providing a mechanistic bridge between upstream signaling abnormalities and the disease-specific phenotypes discussed in Section 4 (16, 144). The integration of these cellular interactions and their contribution to the hallmark pathological features of RA, SLE, and SS are visually represented in **Figure 2**.

## 5 Role of CD40/CD40L in autoimmune diseases

### 5.1 Rheumatoid arthritis

#### 5.1.1 Synovial CD40/CD40L signaling and stromal–myeloid activation

Rheumatoid arthritis (RA) is a long-lasting inflammatory condition that affects approximately 1% of the global population and can result in ongoing joint damage if not treated (148). In RA joints, cells of the innate and adaptive immune system infiltrate the synovium and drive local production of Th1/Th17-type cytokines, chemokines and matrix metalloproteinases by monocytes and synovial cells, accompanied by synovial hyperplasia and cartilage–bone degradation (149). CD40 is functionally expressed on smooth muscle fibroblasts from healthy donors and RA patients as well as on RA synovial cells, and is upregulated by pro-inflammatory mediators such as IFN-γ and TNF (150). Ligation of CD40 on these cells promotes fibroblast proliferation, adhesion molecule expression and secretion of IL-6, GM-CSF and MIP-1, thereby amplifying the inflammatory microenvironment (150). Within co-culture models, activated T cells derived from RA patients stimulate fibroblast-like synoviocytes to produce IL-15, TNF, IL-17, IL-8, and MCP-1 through a CD40-dependent mechanism; likewise, comparable inflammatory interactions are detected upon culturing CD40-activated monocytes with synovial fibroblasts (151, 152). These findings support a model in which CD40 signaling in monocytes and synovial fibroblasts establishes a cytokine–chemokine network that contributes to joint destruction (152), consistent with the observation that CD40 activation in fibroblast-like synoviocytes induces RANKL expression and osteoclast-mediated bone resorption (153).

#### 5.1.2 Additional CD40-responsive stromal and antigen-presenting cell subsets

Additional CD40-responsive cell populations have been described in RA. A stromal population resembling nurse cells, located within the bone marrow and synovium and responsible for promoting B-cell survival, increases CD40 expression upon exposure to IFN-γ, though the biological implications of this upregulation have yet to be clarified (154). The adherent portion of synovial tissue cells—which includes macrophages and dendritic cells—releases TNF following CD40 engagement; moreover, TNF derived from dendritic cells directly participates in collagen degradation within ex vivo cultures (152, 155).

#### 5.1.3 CD40-dependent autoantibodies, experimental models and genetic associations

On the humoral side, a subset of RA patients prone to severe disease develops anti-cyclic citrullinated peptide (anti-CCP) antibodies (156). CD40 signaling is required to induce IgM anti-CCP antibody production by B cells from both healthy controls and RA patients, but only B cells from anti-CCP-positive patients spontaneously secrete anti-CCP antibodies ex vivo, suggesting that they have previously received CD40 signals in the synovial compartment (156). On peripheral blood and synovial T cells derived from RA patients, CD40L expression is induced both more quickly and to a greater extent compared with healthy controls; this molecule promotes immunoglobulin production by B cells and is essential for IL-12 secretion by synovial dendritic cells and macrophages. Furthermore, elevated CD40L expression on T cells is associated with increased disease activity and reduced rates of remission (157). Experimental arthritis models further support a pathogenic role of CD40/CD40L: antagonistic anti-CD40L mAb prevents or ameliorates disease when given before collagen-induced arthritis (CIA) induction or at the time of pathogenic autoantibody transfer in the K/BxN model, but does not reverse established arthritis (158), and transfer of serum from arthritic K/BxN mice into CD40L^−/−^ recipients still induces severe arthritis (159), whereas agonistic anti-CD40 Abs exacerbate CIA with increased IFN-γ production by collagen-specific T cells (160). Genome-wide studies have linked the CD40 locus to juvenile RA (161) and identified a CD40 SNP associated with RA incidence in European but not Korean populations, as well as susceptibility variants in TNFAIP3 (A20) and TRAF1-C5, where TRAF1 cooperates with TRAF2 to enhance CD40 signaling (162, 163). A 3′UTR CD40L polymorphism (24CAs) is under-represented in female RA patients and may exert a protective effect, although its functional basis remains unclear (164).

### 5.2 Systemic lupus erythematosus

#### 5.2.1 Systemic CD40/CD40L activation and B-cell dysregulation

SLE represents a systemic autoimmune disorder characterized by the formation of immune complexes comprising autoantibodies directed against dsDNA and additional nuclear constituents; these complexes deposit within small vessels across the organism, leading to involvement of the skin, joints, lungs, heart, brain, and kidneys (165). Patients exhibit heterogeneous clinical manifestations, and fulfillment of 4 out of 11 criteria is sufficient for diagnosis (165). In active SLE, CD40L is overexpressed on CD4^+^ and CD8^+^ T cells and ectopically expressed on B cells (166). B-cell CD40L is functionally important, as CD40L^+^ B cells from SLE patients spontaneously produce antibodies *in vitro* in a CD40L-dependent manner (166), and transgenic expression of CD40L on B cells is sufficient to induce an age-dependent lupus-like disease in mice (167). B-cell depletion with the anti-CD20 mAb rituximab reduces the proportion of residual B cells expressing CD40 and of T cells expressing CD40L, suggesting that B-cell depletion may partly attenuate CD40/CD40L pathway activation (168). However, although rituximab was mechanistically expected to be beneficial in SLE, major randomized clinical trials did not meet their primary endpoints (169, 170). These findings suggest that attenuation of CD40/CD40L-associated immune activation does not necessarily translate into consistent clinical benefit in SLE. Consistent with systemic activation of this axis, SLE patients exhibit elevated serum levels of soluble CD154 (sCD154), which correlate with disease activity (171).

#### 5.2.2 Target-organ involvement and hematopoietic effects

Target tissues also integrate CD40/CD40L signals in SLE. Human mesangial cells derived from kidney tissue constitutively display CD40 and elevate its expression following stimulation with IFN-γ or activated CD40L-positive platelets obtained from individuals with SLE; moreover, engagement of CD40 on these cells stimulates both cellular expansion and TGF-β release, an effect that may contribute to the development of lupus nephritis (172). In the bone marrow, SLE patients have a reduced frequency and increased apoptosis of CD34^+^ hematopoietic progenitors; CD40 signaling in these cells induces Fas expression and Fas-mediated apoptosis, which may underlie the pancytopenias commonly observed in lupus (173).

#### 5.2.3 Experimental lupus models and CD40 functional variants

In murine lupus models such as (NZB×NZW)F_1_ and (SWR×NZB)F_1_ mice, treatment with anti-CD40L antibodies before disease onset delays or prevents proteinuria, prolongs survival, ameliorates or prevents nephritis and reduces anti-DNA antibody titers, although antibody levels usually rebound after treatment cessation (174). When administered after moderate to severe proteinuria has developed, anti-CD40L mAb still improves survival, renal pathology and immune complex deposition (175). A brief regimen of anti-CD40L treatment is capable of producing sustained positive outcomes regarding survival, anti-dsDNA antibody levels, and renal pathology, especially when administered in conjunction with CTLA4-Ig (176). However, clinical investigations evaluating anti-CD40L in individuals with SLE have generated inconsistent findings (177).

Genetic linkage analyses in humans and mice have identified multiple loci associated with SLE susceptibility (165). The CD40 gene lies on chromosome 20q11.2–13.1, a region suggested to be linked to SLE incidence. A missense SNP, rs11086998 G, introduces a P227A substitution in the cytoplasmic tail of CD40 near the TRAF6-binding site and has been reported at higher frequency in individuals of Native American ancestry, including those of Mexican and South American descent (178). Although rs11086998 does not show a clear association with SLE susceptibility in Hispanic populations (178), Hispanic patients overall tend to have more severe disease manifestations, particularly lupus nephritis (179). From a functional standpoint, the CD40-P227A variant exhibits enhanced signaling relative to wild-type CD40, resulting in elevated production of antibodies and pro-inflammatory cytokines, a phenomenon attributed to hyperactivation of the JNK pathway (178). This variant may therefore contribute to disease severity or flares in individuals who have already developed SLE (178), while the overall contribution of CD40 polymorphisms to SLE and other autoimmune diseases across different populations remains to be clarified, as association studies and meta-analyses have yielded heterogeneous results (180, 181).

### 5.3 Sjögren’s syndrome

#### 5.3.1 Glandular pathology and local CD40/CD40L signaling

SS represents a persistent autoimmune condition characterized principally by involvement of the exocrine glands, resulting in clinical manifestations including xerostomia and xerophthalmia (182). It affects approximately 0.1-0.4% of the population, with a higher prevalence in females compared to males, at a ratio of approximately 9:1 (183). SS frequently coexists with other autoimmune conditions, including SLE and RA, and has been linked to an elevated risk of lymphoma development (184). The disease is characterized by lymphocytic infiltration in the exocrine glands, a significant increase in B cell numbers, and a breakdown of normal immune tolerance (185).

In SS, the CD40–CD40L signaling axis is critically involved in driving both B-cell and T-cell activation (186). CD40, present on the surface of B cells, macrophages, and dendritic cells, engages with its ligand CD40L—a molecule found predominantly on activated T cells (16). This molecular interplay is indispensable for effective antigen presentation and the subsequent induction of T cell-dependent immune responses, processes that lie at the core of SS pathogenesis (186). Both CD40 and CD40L are upregulated in the salivary glands of SS patients, promoting chronic inflammation and immune activation (187). Additionally, studies have shown that higher levels of soluble CD40L (sCD40L) are detectable in the serum of SS patients, and this CD40/CD40L-driven activation has been linked to systemic disease activity and chronic glandular inflammation (188).

#### 5.3.2 Genetic associations and preclinical CD40/CD40L blockade

At the genetic level, polymorphisms in genes involved in the CD40/CD40L pathway have been associated with susceptibility to several autoimmune diseases, and functional CD40 variants (such as rs1883832 and rs4810485) have been shown to modulate CD40 expression on B cells and monocytes, suggesting that similar mechanisms may also contribute to genetic risk in SS (189). A specific C/T single nucleotide polymorphism (SNP) in the CD40 gene has been linked to higher CD40 expression, which may enhance immune responses and promote disease progression (190). In particular, the CC genotype of this polymorphism is associated with higher CD40 expression on B cells (190). In addition, CD40 is strongly expressed on salivary gland epithelial cells in SS lesions (191), which together may drive autoimmune responses in SS patients. Blocking the CD40/CD40L interaction with antagonistic antibodies has shown promise in SS experimental models, reducing B cell activation, inflammatory cell infiltration, and tissue damage (192).

Functionally, blockade of CD40L in mouse models of Sjögren’s syndrome has prevented disease progression and tissue damage, indicating that the CD40–CD40L interaction is a key mediator of immune dysregulation in SS (192). Furthermore, therapies targeting CD40 or CD40L, such as monoclonal antibodies, are currently being studied in clinical trials, and early results suggest they hold promise for improving disease outcomes and reducing systemic inflammation (145).

## 6 Therapeutic strategies targeting the CD40/CD40L interaction

### 6.1 CD40/CD40L pathway modulation in rheumatoid arthritis

#### 6.1.1 Fc-free CD40L blockade with VIB4920

VIB4920 (MEDI4920) is a novel CD40L-binding protein derived from two Tn3 proteins based on fibronectin type III domains, fused to human serum albumin (66). VIB4920 targets CD40L but lacks an Fc domain, making it less likely to induce thromboembolic complications (66). A Phase 1 randomized, blinded, placebo-controlled, single-dose escalation trial evaluating VIB4920 reported favorable safety and tolerability profiles in healthy adult participants (66). In a subsequent Phase 1b randomized, double-blind, placebo-controlled, multiple-dose escalation study, administration of VIB4920 to patients with RA led to a significant reduction in disease activity by day 85, as assessed by DAS28-CRP scores, alongside improvements in additional clinical parameters—including tender and swollen joint counts, C-reactive protein (CRP) levels, as well as global patient and physician evaluations (66). These clinical benefits were associated with substantial decreases in rheumatoid factor and other circulating markers linked to RA disease activity, such as ACPA, soluble CD40L, and CXCL13 (193).

Clinical studies of VIB4920 (dazodalibep) have reported anti-drug antibody formation, suggesting that immunogenicity may represent an important consideration for future CD40L-targeted therapies (66, 194). Although these antibodies did not clearly abrogate clinical responses within the study period, their long-term clinical significance remains to be further determined (194). Despite these immunogenicity considerations, a phase 2 randomized, double-blind, placebo-controlled trial in rheumatoid arthritis (MIDORA; NCT04163991) has evaluated the efficacy and safety of VIB4920 (dazodalibep), showing significant reductions in disease activity with an acceptable tolerability profile (193).

#### 6.1.2 Non-depleting anti-CD40 monoclonal antibody BI-655064

BI-655064 is a humanized antagonistic anti-CD40 IgG1 monoclonal antibody engineered to minimize Fc-mediated effector functions (195). In a phase 2 trial in rheumatoid arthritis, BI-655064 reduced activated B-cell subsets, but the overall efficacy signal was not statistically convincing, as the difference in ACR20 response versus placebo did not reach conventional significance (196). This limitation was further reflected in lupus nephritis studies, in which BI-655064 failed to demonstrate a dose–response relationship for the primary efficacy endpoint (197). Taken together, BI-655064 showed target engagement and measurable immunological activity, but these effects did not translate into convincing or consistent clinical efficacy across indications, and the available evidence did not support clear therapeutic success.

### 6.2 CD40L-targeting therapeutics: from early antibodies to next-generation approaches

#### 6.2.1 First-generation Fc-competent anti-CD40L antibodies: ruplizumab and toralizumab

Ruplizumab (Hu5c8, BG9588) is a humanized IgG1 monoclonal antibody specific to CD40L, one of the first molecules developed to target the CD40 pathway (177). In an open-label Phase 2 study of patients with proliferative lupus nephritis, ruplizumab significantly reduced anti-dsDNA antibody levels and hematuria, while also increasing complement C3 concentrations (177). Furthermore, there was a reduction in the number of CD38-high expressing B cells and plasma cells that secrete IgM and IgG anti-dsDNA antibodies (198). In that study, decreases in anti-dsDNA titers were accompanied by improvements in SLE disease activity indices (177, 198), which is consistent with broader biomarker data showing that anti-dsDNA, anti-nucleosome, anti-C1q and anti-histone antibodies correlate with lupus nephritis activity and global SLEDAI scores (199). However, despite these promising clinical results, further development of ruplizumab was halted due to cardiovascular thromboembolic events (TEs) (200). After administering ruplizumab to rhesus monkeys, thrombotic pathological findings, including pulmonary vascular thrombosis and vascular lesions, were observed (201).

Toralizumab (IDEC-131) represents a humanized monoclonal antibody directed against CD40L, which has undergone assessment in several early-stage clinical investigations—comprising Phase 1 and Phase 2 trials involving patients with systemic lupus erythematosus (202, 203). Despite doses ranging from 0.05 to 15.0 mg/kg, toralizumab (IDEC-131) demonstrated a favorable safety and tolerability profile without adverse events (202, 203). Unfortunately, no efficacy advantage was demonstrated when IDEC-131 was compared to placebo in patients with mild-to-moderate disease activity (202). Like ruplizumab, IDEC-131’s further development was terminated due to increased thromboembolic events observed in other trials (200).

#### 6.2.2 Fc-free/Fc-modified CD40L blockade: dapirolizumab pegol and Fab-based strategies

The thromboembolic events observed with ruplizumab and toralizumab appear to depend on the presence of a functional Fc fragment (16). Upon activation, platelets display CD40L on their surface; moreover, *in vitro* studies have demonstrated that immune complexes formed between soluble CD40L and anti-CD40L monoclonal antibodies are capable of inducing platelet aggregation (16, 204). Inhibiting platelet Fc receptors can block this antibody-mediated platelet aggregation (204).

One method of reducing thromboembolic events is by eliminating the Fc fragment, which led to the development of dapirolizumab pegol (CDP7657), a PEGylated anti-CD40L Fab’ fragment (201). This agent has shown no evidence of thromboembolic complications in preclinical studies (201). In Phase 1 studies, dapirolizumab pegol demonstrated no severe treatment-related adverse events. Exploratory analyses in patients with high baseline disease activity suggested that this drug has potential for clinical improvement, including a reduction in anti-dsDNA antibodies and downregulation of genes associated with B-cell and plasma cell activation (205). Dapirolizumab pegol is not the only Fab-based CD40L-targeting strategy. Another anti-CD40L Fab fragment, Fab20, has recently been reported in preclinical studies to block CD40–CD40L interaction and suppress human B-cell activation and differentiation, further supporting Fc-free ligand blockade as a potentially safer design strategy, although current evidence remains preclinical (206).

#### 6.2.3 Other emerging CD40L-targeting therapeutics

Beyond dapirolizumab pegol, several additional CD40L-targeting agents have expanded the therapeutic landscape and illustrate how the field has shifted toward Fc-sparing or Fc-modified strategies. Frexalimab (SAR441344), a next-generation anti-CD40L antibody engineered to reduce Fc-mediated platelet activation, is among the most clinically advanced agents in this class. It showed positive phase 2 results in relapsing multiple sclerosis and has progressed into phase 3 evaluation, highlighting the continued therapeutic interest in safer CD40L blockade (207).

Additional molecules have also broadened development efforts in this area. Tegoprubart (AT-1501), an Fc-modified anti-CD40L antibody developed primarily in transplantation, prolonged graft survival and supported graft function in nonhuman primate islet and kidney transplantation models, supporting its immunomodulatory potential while avoiding the thromboembolic liabilities that limited earlier agents (208). TNX-1500, an Fc-modified anti-CD154 antibody derived from the hu5c8/ruplizumab lineage and developed mainly in transplantation-oriented preclinical studies, was not associated with platelet activation *in vitro* and consistently inhibited kidney allograft rejection *in vivo*, further supporting the feasibility of safer CD40L blockade through Fc engineering (209).

Other next-generation approaches remain at earlier stages. Letolizumab (BMS-986004) represents another Fc-silent/Fc-modified CD40L-targeting approach; however, publicly available efficacy data remain limited, and its clinical role is not yet clearly defined (210). Taken together, these agents show that CD40L-targeting development has moved beyond the earliest Fc-competent antibodies and now increasingly focuses on preserving target engagement while reducing Fc-mediated platelet-related toxicity.

### 6.3 Iscalimab as a non-depleting CD40 blocker: from transplantation to Sjögren’s syndrome

#### 6.3.1 Non-depleting anti-CD40 antibody iscalimab in transplantation

Iscalimab (CFZ533) is a human non-agonistic anti-CD40 monoclonal IgG1 antibody that blocks CD40. It contains a modified Fc domain that prevents it from mediating Fcγ-dependent effector functions, thus rendering it non-depleting (211). In preclinical studies, iscalimab was shown to reduce humoral responses and germinal center (GC) formation in monkeys following kidney transplantation (212).

In Phase 1/2 studies, iscalimab, in combination with mycophenolate mofetil (MMF) and corticosteroids (CS), showed preliminary efficacy in kidney transplantation. Furthermore, early reports suggested potential benefits in renal function and favorable allograft histology (213, 214).

#### 6.3.2 Iscalimab in primary Sjögren’s syndrome and CXCL13 as a biomarker

In a phase 2a trial in primary Sjögren’s syndrome, iscalimab showed dose-dependent pharmacodynamic effects but mixed clinical findings (215). No clear treatment effect was observed in the low-exposure subcutaneous cohort, whereas the higher-dose intravenous cohort showed a signal of clinical improvement, including a significant reduction in disease activity and directional improvement in several secondary measures (215). Iscalimab was also associated with reduced CXCL13 levels, suggesting target engagement and a possible effect on germinal center-related mechanisms, although biomarker changes alone cannot establish durable clinical efficacy (215–217). Overall, the clinical evidence remained inconsistent across cohorts and did not establish sufficiently robust efficacy to support clear therapeutic success. These findings illustrate a broader challenge in CD40-pathway targeting: target engagement does not necessarily translate into reproducible clinical benefit.

### 6.4 Horizontal comparison and summary of clinical candidate drugs

Therapeutic strategies targeting the CD40/CD40L axis have evolved from early approaches based on complete antibody blockade to more precise and safer context-selective modulation. To facilitate a clearer comparison of the design strategies and clinical performance of different candidate agents across major autoimmune diseases, a consolidated comparison of the principal drugs discussed in this review is provided in **Table 1**.

**Table 1:** Representative therapeutic candidates targeting the CD40/CD40L pathway and their key characteristics.

Drug	Target	Molecular design	Main indications	Key efficacy and safety observations
VIB4920	CD40L	Fc-free Tn3 fusion protein based on fibronectin type III domains.	RA, SS	Showed early reductions in disease activity and CXCL13 levels; Fc-free design may reduce thromboembolic risk, although longer-term efficacy remains to be established.
Iscalimab (CFZ533)	CD40	Non-depleting, non-agonistic anti-CD40 monoclonal antibody with Fc modifications	SS, Transplantation	Produced early pharmacodynamic and biomarker signals, with clinical improvement limited to selected cohorts; broader development did not establish consistent efficacy or clear therapeutic success.
BI-655064	CD40	Antagonistic anti-CD40 monoclonal antibody engineered to reduce Fc-effector functions	RA	Showed target engagement and reduced activated B-cell subsets, but did not demonstrate convincing overall clinical efficacy, and later evaluation did not establish clear therapeutic success.
Dapirolizumab pegol	CD40L	PEGylated anti-CD40L Fab’ fragment.	SLE	Showed immunomodulatory activity and favorable early safety findings, with no thromboembolic signal reported in early-phase studies.
Ruplizumab (Hu5c8)	CD40L	First-generation humanized anti-CD40L IgG1 monoclonal antibody	SLE	Showed biological activity, including reduction of autoantibody-related markers, but development was halted because of thromboembolic events.
Toralizumab (IDEC-131)	CD40L	First-generation humanized anti-CD40L monoclonal antibody	SLE	Did not demonstrate sufficient clinical efficacy, and development of this class was further constrained by thromboembolic safety concerns.
Frexalimab (SAR441344)	CD40L	Next-generation anti-CD40L monoclonal antibody	Multiple sclerosis	Showed positive phase 2 results in relapsing multiple sclerosis and has progressed into phase 3 evaluation, making it one of the most clinically advanced CD40L-targeting agents currently reported.
Tegoprubart (AT-1501)	CD40L	Fc-modified anti-CD40L antibody	Transplantation	Prolonged graft survival and supported graft function in nonhuman primate islet and kidney transplantation models; designed to mitigate thromboembolic risk associated with earlier anti-CD40L antibodies.
TNX-1500	CD154/CD40L	Fc-modified anti-CD154 antibody derived from the hu5c8/ruplizumab lineage	Transplantation	Was not associated with platelet activation *in vitro* and consistently inhibited kidney allograft rejection *in vivo* in preclinical studies.
Letolizumab (BMS-986004)	CD40L	Fc-silent/Fc-modified CD40L-targeting approach	Early development/transplantation-related settings	Designed to reduce Fc-mediated safety liabilities; publicly available efficacy data remain limited.
Fab20	CD40L	Fab fragment	Preclinical	Preclinical Fab-based strategy reported to block CD40–CD40L interaction and suppress human B-cell activation and differentiation.

## 7 Conclusion

This review systematically summarizes the role of the CD40/CD40L axis in autoimmune diseases from three perspectives: signal transduction mechanisms, disease pathogenesis, and clinical therapeutic interventions. Mechanistically, this pathway activates multiple downstream signaling cascades, including NF-κB, MAPK, and PI3K/Akt, through TRAF-dependent signaling. Notably, these signaling events exhibit pronounced heterogeneity across different cell types, providing a structural basis for diverse immune responses. In rheumatoid arthritis, systemic lupus erythematosus, and Sjögren’s syndrome, dysregulated CD40/CD40L signaling plays a central role in ectopic germinal center formation, pathogenic autoantibody production, and chronic tissue inflammation.

Emerging therapeutic strategies targeting CD40 or CD40L further support the feasibility of this axis as a therapeutic target in autoimmune diseases. However, these advances also highlight the need to carefully balance therapeutic efficacy with safety, particularly with regard to thromboembolic risk. Future studies should focus on biomarker-guided patient stratification and context-selective modulation of CD40 signaling. Through the rational design of next-generation therapeutics that exploit cell type-restricted signaling nodes while minimizing Fc-mediated adverse effects, it may be possible to achieve durable disease control within an acceptable safety profile.
